# P-1841. Characterizing Hepatitis B Virus Surface Antigen Positivity in United States Service Members

**DOI:** 10.1093/ofid/ofaf695.2010

**Published:** 2026-01-11

**Authors:** Shelby Hill, Jessica Basso, Samuel Owen, Joseph Marcus

**Affiliations:** San Antonio Uniformed Services Health Education Consortium, San Antonio, TX; San Antonio Uniformed Services Health Education Consortium, San Antonio, TX; Brooke Army Medical Center, San Antonio, Texas; Brooke Army Medical Center, San Antonio, Texas

## Abstract

**Background:**

Hepatitis B virus (HBV) leads to significant morbidity and mortality but is frequently a subclinical infection. The United States military does not screen service members for HBV exposure prior to accession. While universal exposure screening with HBV surface antigen (HBVsAg) has been proposed, there is limited data on service members who test positive for HBVsAg. This study aims to better characterize service members with positive HBVsAg.

**Table 1:**
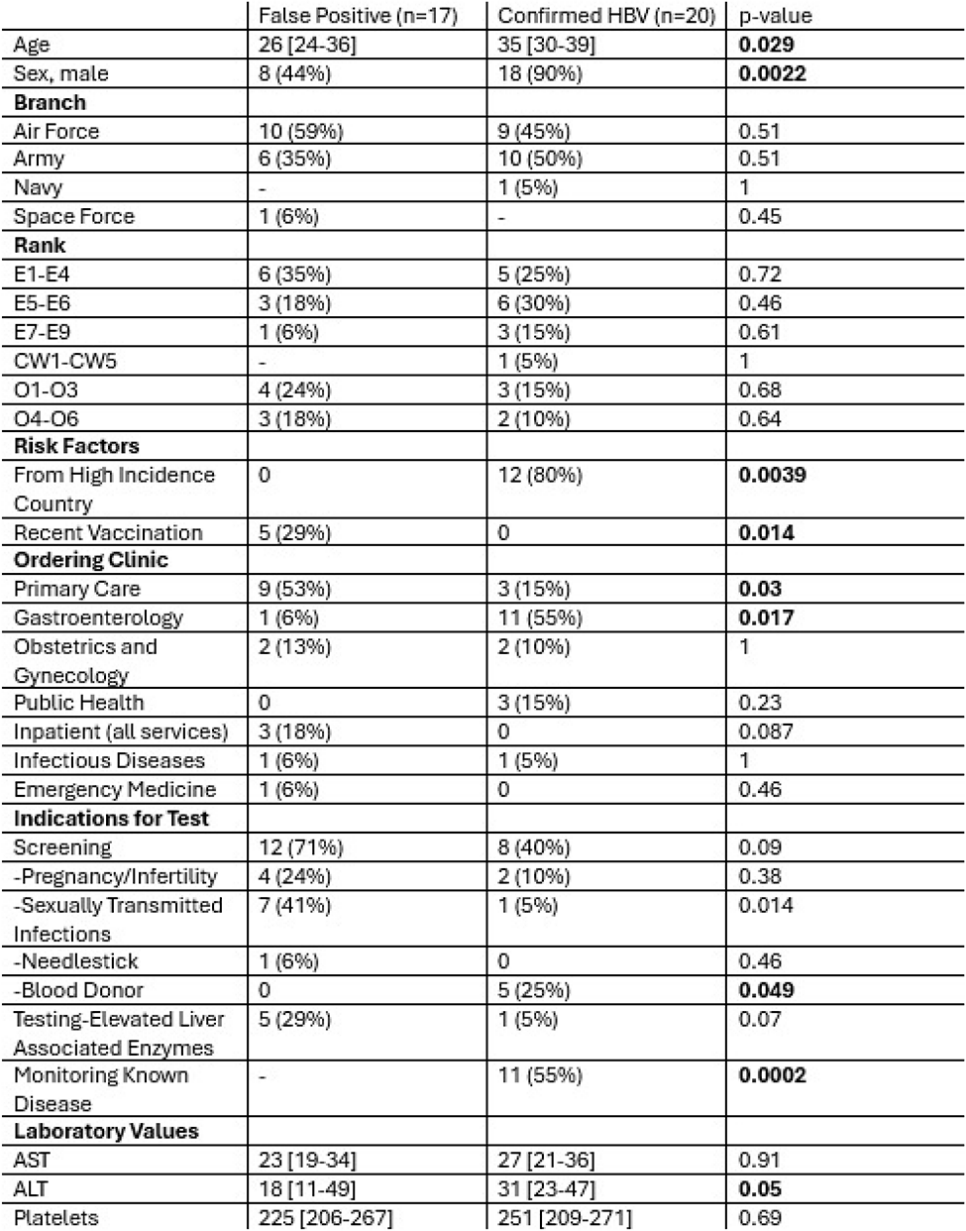
Characteristics of active-duty military service members with a positive hepatitis B virus surface antigen test, 2022-2024

**Methods:**

We analyzed all service members with a positive HBVsAg at a large military base between January 2022 and May 2024. We reviewed associated HBV serologies in these patients and determined final HBV diagnosis. We then compared factors associated with a true infection versus a false positive test.

**Results:**

During the study period, 39 active-duty service members had a positive HBVsAg. Of those, 37 (95%) had adequate follow-up testing to determine their HBV status. Service members tested were predominantly male (69%), junior enlisted (67%), and were tested as part of a screening protocol (56%). Further testing revealed 18 (46%) patients with chronic HBV, 2 (5%) patients with acute HBV, and 17 (44%) false positives. There were several differences in demographics, testing locations, risk factors, and laboratory values in patients with true disease as compared to false positives (Table 1). Notably, no patient from a high incidence country (80% vs. 0%, p=0.004) had a false positive, and no patient with a recent HBV vaccine (29% vs. 0%, p=0.02) had true disease.

**Conclusion:**

HBVsAg screening has been proposed in the military, but current testing practices are unknown. In this analysis of a large, multi-service military base, screening was the most common indication for identifying a positive HBVsAg; however, false positives were common. If a large-scale screening protocol is established in the military, it should focus on screening patients with identified risk factors and be temporally separated from HBV vaccination efforts. These measures should increase the likelihood that a positive HBVsAg represents true infection, thereby minimizing unnecessary testing and consults, and increasing cost effectiveness of universal screening.

**Disclosures:**

All Authors: No reported disclosures

